# The Sphygmometer and Aortic Regurgitation

**Published:** 1915-06-05

**Authors:** 


### THE SPHYGMOMETER AND AORTIC REGURGITATION.

### An Opportunity for Valuable Work.

The sphygmometer is an apparatus which is now fairly widely employed in clinical practice, and, though it has perhaps failed to display all the virtues originally claimed by its sponsors, it has a quite distinct field of usefulness. There are still some disputes in reference to the manner of stating its records, as also in reference to the diagnostic and prognostic significance of these. Thus it is strongly held by one school that a large element in determining the height of the index is the resistance of the arterial wall, as distinct from the intra-arterial pressure of the blood. This resistance, too, it is said, is determined not merely by degenerative changes, but by the muscular elements of the vessel. Contraction of these latter will offer considerable resistance to pressure, while with relaxation this resistance will be diminished. Hence in one and the same patient the sphygmometer reading may vary widely at different times in consequence merely of a change in the state of the muscular coat of the brachial artery. Opposed to all this is the view that the resistance of the arterial wall is a negligible factor, and that the sphygmometer record may be accepted as an approximately accurate picture of the state of the blood pressure. This difference of opinion is serious enough as a matter of doctrine, but is of less importance in practice, seeing that most observers agree that abnormally high records usually mean those cardio-vascular conditions which tend to premature degeneration and to fibrotic changes in the kidneys. The difference is not completely resolved by this statement, but, broadly, the conclusion thus presented is generally accepted. There is one proposal arising from the discussion which might be more generally noted. It is that the measurements obtained by the sphygmometer should be stated as such, and should not be presented in terms of blood pressure. The sphygmometer figures are facts. Whether they certainly mean blood pressure is a matter of opinion. And descriptive scientific terms ought to represent facts only, and ought not to include speculations or hypotheses.

In the main, clinical observations and the discussions arising therefrom have been concentrated on the maximum measurements registered by the sphygmometer. Obviously there might be advantages in obtaining a minimum record, and especially on the view that the measurement is concerned with the state of the blood pressure. There is a very simple method of doing this, though it does not appear to be generally known. All that is necessary is, when the armlet of the sphygmometer is in position, to put the bell of the ordinary binaural stethoscope over the brachial artery a little below the lower border of the armlet. When pressure is raised so as to occlude the vessel no sound is heard; but, as the pressure is gradually lowered, there comes a point where a knocking or thudding sound is produced with each cardiac systole. This point is to be taken as the maximum sphygmometer reading, or, in current phrase, the maximum or systolic blood pressure. Next the sphygmometer pressure is further lowered, and for a time the knocking noise continues until a point is reached where it abruptly ceases. This second point is taken to indicate the minimum or diastolic blood pressure, though it would perhaps be more advisable to name it the minimum sphygmometer reading. The physiological explanation of these events is not easy. The brief fact is that by the auditory method it is possible to get a certain maximum and a certain minimum record of measurement.

It is here that the relation of the sphygmometer to aortic regurgitation arrives on the scene. In normal persons, and also in the vast majority of those who appear as patients, the maximum and minimum records above indicated show corresponding features. If one is high, so is the other, and with a low maximum a low minimum is to be expected. The difference between them, often called the pulse pressure, may range from, say, 30 to 100 mm.; and the minimum record, as a matter of experience, is rarely less than 50 mm. To this, aortic regurgitation offers a striking exception. Here the difference between maximum and minimum may reach 150 mm. or more, and the minimum record may decline to 20, 10, or even 0. It is doubtful if any condition other than aortic regurgitation will produce these low records. At least this may be said, that such records associated with a high maximum reading can hardly mean anything but aortic regurgitation. Less confidently, perhaps, but still with a fairly firm conviction, any wide disparity between maximum and minimum records suggest the same diagnosis. The observation is of obvious value, especially when doubt arises as to the interpretation of an ambiguous murmur. Yet at a recent medical meeting such an issue was warmly debated, and no one of the speakers, to judge from the report, thought an appeal to the sphygmometer was worth consideration.

All this is not to say that in every case of aortic regurgitation the maximum sphygmometer record will run down to the low levels above stated. It is therefore quite possible to find the auscultatory and other evidences of regurgitation and yet with these no depression of the sphygmometer record. What is the significance of these differences? It is at least possible, as has just been hinted, that the contrast depends on the extent of the regurgitation. Hence it may be that the sphygmometer in aortic regurgitation has a prognostic as well as a diagnostic function. A low minimum record suggests a severe case, and the absence of this a relatively favourable one. To establish this conclusion securely further observations are needed, and these might well engage the attention of practitioners who are able to watch patients over long periods of time. Here is an opportunity for an interesting and valuable application of the sphygmometer.

